# The Need for Clinical Decision Support Integrated with the Electronic Health Record for the Clinical Application of Whole Genome Sequencing Information

**DOI:** 10.3390/jpm3040306

**Published:** 2013-12-18

**Authors:** Brandon M. Welch, Kensaku Kawamoto

**Affiliations:** 1 Program in Personalized Health Care, University of Utah, 15 North 2030 East, EIHG Room 2110, Salt Lake City, UT 84112, USA; 2 Department of Biomedical Informatics, University of Utah, 26 South 2000 East, Room 5775 HSEB, Salt Lake City, UT 84112, USA; E-Mail: kensaku.kawamoto@utah.edu

**Keywords:** clinical decision support systems, medical genetics, genomics, genetic testing, electronic health records, health information technology, personalized medicine

## Abstract

Whole genome sequencing (WGS) is rapidly approaching widespread clinical application. Technology advancements over the past decade, since the first human genome was decoded, have made it feasible to use WGS for clinical care. Future advancements will likely drive down the price to the point wherein WGS is routinely available for care. However, were this to happen today, most of the genetic information available to guide clinical care would go unused due to the complexity of genetics, limited physician proficiency in genetics, and lack of genetics professionals in the clinical workforce. Furthermore, these limitations are unlikely to change in the future. As such, the use of clinical decision support (CDS) to guide genome-guided clinical decision-making is imperative. In this manuscript, we describe the barriers to widespread clinical application of WGS information, describe how CDS can be an important tool for overcoming these barriers, and provide clinical examples of how genome-enabled CDS can be used in the clinical setting.

## 1. Introduction

Whole genome sequencing (WGS) is on the cusp of revolutionizing medicine. In the decade since the completion of the Human Genome Project, advancements in sequencing technology have made it feasible to sequence a patient’s entire genome for clinical uses [1,2]. Indeed, many patients have already had their genome sequenced for direct clinical application to date [3,4,5,6]. While most of these cases have been for rare, undiagnosed diseases, or in the context of clinical research, it will not be long before WGS information is available for routine medical care on a widespread scale. This will further enable the practice of personalized medicine, which has the potential to reduce costs and improve the quality of care [7,8].

The WGS information can be used to support clinical diagnosis, direct preventative efforts, and guide therapeutic decisions in the clinic. Indeed, the clinical use of WGS information may hold several advantages over current genetic testing practices:

- There are nearly 3,000 diseases for which individual genetic tests are available [9]. As clinicians pursue a clinical diagnosis today, they sometimes must order several single gene tests until a particular diagnosis is either confirmed or rejected. This process may take a significant amount of time and money as individual genetic tests can cost anywhere between hundreds to thousands of dollars. However, with the ability of WGS to ascertain the results for thousands of available genetic tests at once, it may become financially beneficial and more efficient for clinicians and payers to recommend WGS in lieu of single gene tests, as the diagnostic odyssey and associated costs could be reduced [10].
- Genetic tests are often ordered today as a result of a clinical indication; examples of clinical indications include particular phenotypes, family history, or preliminary diagnosis [11]. This approach is also reinforced by some health insurance providers who require clinical indication and prior authorization in order for certain genetic tests to be reimbursed [12]. However, such an approach can hinder the effective use of genetic information for decision-making, particularly for preemptive and preventative care where clear clinical indications may not always be present [13]. Indeed, if a clinical indication is not present at the time of assessment or clinicians are unaware that a particular genetic test is available, they may miss an opportunity to order the genetic test at a time that can add value to a clinical scenario. Nevertheless, with a patient’s WGS information available and readily accessible throughout a patient’s life, genetic information can be leveraged for preemptive and preventative care to a larger extent than it iscurrently.

As WGS is not widely used in the clinical setting at this point, these examples represent theoretical advantages over current genetic testing practices. Until clinical and outcomes research studies on WGS can confirm or reject the validity of these scenarios, these examples will continue to remain theoretical.

Nevertheless, to be most effective, WGS will almost certainly require the effective use of clinical decision support (CDS) integrated into the clinical workflow. However, a systematic review by the authors on the use of CDS for genetically-guided personalized medicine found a significant lack of system descriptions or research studies on the use of CDS to support the clinical use of WGS information [14]. A number of studies identified in the systematic review, as well as several recent papers and research efforts have described CDS systems that integrate genetics information with CDS. However, these solutions are generally limited in scope with regards to the genetic information used (generally not necessarily WGS), are not integrated within the electronic health record (EHR), or are implemented using CDS approaches that are difficult to scale [15,16,17,18]. Indeed, research on CDS solutions for WGS information in particular is still very nascent [18]. While some principles from these efforts can be translated to scalable WGS CDS approaches, additional capabilities will be necessary for the consistent and widespread adoption of CDS capabilities for WGS information [19]. Thus, in order to help establish a foundation for future research and development of scalable CDS for WGS information, this manuscript makes the case for CDS for the WGS. To begin making this case, we start by outlining the many barriers to the effective clinical application of WGS information.

## 2. Barriers to Effective Clinical Application of WGS Information

Significant barriers exist for the effective and efficient application of WGS information in routine clinical care. These barriers, which will each be described in further detail, include current laboratory reporting methods, the complexity of genetics, the limited physician proficiency in genetics, and the lack of genetics experts. While these barriers have contributed to the slow and inconsistent clinical adoption of genetics [20], we believe that the increased clinical demands as a result of WGS information will make these problems worse.

### 2.1. Static Laboratory Reports Intended for Human Consumption

Typically, genome sequencing, annotation, and variant classification are performed in Clinical Laboratory Improvement Amendments (CLIA)-approved diagnostic laboratories. If these laboratories follow current standard workflow [21], they will send a static test report by mail, fax, or PDF to the treating clinician. While this workflow has met the needs of current pathology and many genetic tests to date, there are several shortcomings to this approach for WGS information. First, there are roughly three million variants (*i.e.*, mutations) per human genome, and this number is too large to be managed on a single static report. Second, as the genome variant knowledge base continues to grow and change, the need to reclassify variants and notify treating clinicians will become necessary. It is recommended that laboratories take responsibility for updating clinicians to changes in variant interpretation [22]; however, this represents a significant, uncompensated workload upon the laboratory if managed manually. For instance, every individual has hundreds of thousands of variants of unknown significance (VUS), which are variants yet to be associated with a phenotype or ruled out as benign [5]. Over a seven year period, one study found that 14.5% of reported variants had to be reclassified, 27% of which were initially VUS [23]. Third, a static genome report document does not support the automatic provision of CDS at the point of care. Ideally, data should be represented in a discrete, standardized, and digital form accessible to computer interpretation. This is not the case in static laboratory reports. As a result, current laboratory reports require a clinician to manually assess and interpret the reports. At the scale of WGS information, such an approach will likely render most of the information ineffective due to massive information overload [24]. 

### 2.2. Complexity of Genetics

Genetics research has brought to light the tremendous complexity of genomic interactions on phenotypes [25]. Within a gene, there can be variants such as point mutations, deletions, insertions, tandem repeats, and splice site mutations, which can all affect the protein product and associated phenotypes. There can be hundreds of possible variants within a specific gene or pathway of genes that contribute to a particular disease etiology. Furthermore, as mentioned previously, not all variants are known to be pathogenic; some are benign while others are VUS [26]. Additionally, gene variants cannot be interpreted in isolation; gene regulatory regions, post-transcriptional modification, transcriptional expression, copy number variations, epistasis, pleiotropy, gene-environment interactions, and other epigenetic influences are additional factors that can modify and impact phenotypes [27]. Relying on a clinician to know all possible genes, variations, and interactions for a particular disease and then to apply that information appropriately at the point of care without assistance is a futile proposition. This is particularly important for common diseases such as heart disease, diabetes, and cancer, which may involve tens to hundreds of contributing genetic, epigenetic, and environmental influences [28]. The interpretation of genetics in the clinic is a complicated endeavor involving numerous genomic interactions and associations which must all be managed accurately for appropriate clinical interpretation.

### 2.3. Limited Physician Proficiency in Genetics

As stated earlier, there are nearly 3,000 diseases for which an individual genetic test is available [9]. It is beyond the capacity of any human to know and manage all known genetic tests, pertinent genetic contributions, disease-causing variants, and relevant family history associations without computerized support [24,29]. To illustrate, there are almost 1,200 known variants within the adenomatous polyposis coli (*APC*) gene, which is associated with a rare, inherited form of colon cancer [30]. Similarly, almost 2,000 variants in the *CFTR* gene are associated with cystic fibrosis [31]. It is impossible for a clinician to know all possible variants and related variant classifications within a single gene, let alone every variant in the approximately 20,000 genes in the entire human genome. Moreover, genetics is a rapidly growing and evolving field of research; clinicians today do not have the capacity to stay up to date on the current and ever expanding genetics knowledge base [32]. It has been found that it can take 15 or more years for “traditional” medical discoveries to be translated from bench to bedside [33]. Due to the exceeding complexity and breadth of genomics, we expect genetics discoveries to take significantly longer to translate to clinical care with much lower success without additional support.

Furthermore, most clinicians received little to no formal training on the application of genetics to clinical practice [34,35,36]. Any training they may have received was likely relatively basic, primarily focused on monogenetic diseases with simple inheritance patterns [37,38]. The training required to analyze the complexities of genomics is beyond the scope of most medical school curricula, which are already burdened with numerous competing demands [39]. Accordingly, physicians rate their knowledge of genetics as ‘fair to poor’ [40,41], with a number of studies confirming their poor knowledge and clinical interpretation of genetics [42,43]. Even education programs specifically designed to teach genetics to clinicians only produce modest results, with substantial gaps in clinician knowledge on how to appropriately apply genetics at the point of care [43,44]. Expecting clinicians to properly manage a patient’s WGS information on their own, even after targeted education, is a daunting proposition.

### 2.4. Lack of Genetics Professionals

In recent decades, medical genetics and genetic counseling are two specialties that have arisen to provide in-depth knowledge and to help manage the complexity of clinical genetics. Medical geneticists are physicians trained to evaluate patients and assess and manage the genetic contribution to diseases [45]. Genetic counselors are masters-level health professionals who work with physicians to help assess genetic risk and communicate genetic information, such as test results, to patients and their families [46]. While these specialties attempt to fill the need, there are only 1,200 medical geneticists and 3,000 certified genetic counselors who are unequally and insufficiently distributed across the United States today [47,48]. Today, a genetic professional typically spends seven hours preparing for and meeting with new patients [49]. With only one medical geneticist per 262,000 U.S. citizens and one genetic counselor per 105,000 U.S. citizens, each genetic professional would have to work for over 239 years if every person in the U.S. had their genome sequenced today [50]. The insufficient supply of genetics experts is unlikely to change in the foreseeable future, as aspiring clinicians are not entering the genetics profession at the rate needed for growth [51]. Furthermore, with the potential for genetics to impact so many clinical decisions [52], and the labor intensive nature of genetic interpretation and counseling [49], it will be inadequate, inefficient, and cost-prohibitive to have a genetics professional available every time genetic information is used at the point of care [53].

## 3. CDS as a Solution

Clearly, significant barriers exist which will hinder the effective and efficient application of genetics at the point of care. Nevertheless, a number of thought leaders and researchers have recognized this problem and have identified EHRs incorporating CDS as a practicable solution to help clinicians manage the complexities of genetics at the point of care [53,54,55,56,57]. CDS entails providing clinicians, patients, and other healthcare stakeholders with pertinent knowledge and/or person-specific information, intelligently filtered or presented at appropriate times, to enhance health and healthcare [58]. Examples of CDS include medication dosing support, order facilitators, point of care alerts and reminders, relevant information display, expert systems, and workflow support [59]. Research on CDS has been conducted for decades and is a proven solution for assisting clinicians in providing appropriate care and reducing errors in many clinical use cases [60,61,62,63]. Furthermore, CDS has the ability to translate knowledge from bench to bedside much more efficiently than traditional methods [54]. As a result of this success, the Office of the National Coordinator for Health IT (ONC) has announced that CDS will be a key component of proposed Meaningful Use Stage 3 criteria, which are expected to be proposed in 2014 [64].

### 3.1. Overcoming WGS Barriers

CDS has been identified as a potential solution for supporting WGS information in the clinic because of its ability to overcome many of the barriers to effective use of WGS information described above. CDS has the capacity to process complex, disparate clinical data and present actionable, evidence-based recommendations in a way that is usable by a clinician at the point of care [63]. This capability will be essential for CDS for WGS information because certain clinical use cases may require the combination of several genetic loci as well as the patient’s health history, family history, and environmental influences to develop accurate clinical assessments and recommendations. Furthermore, CDS is able to automate the application of complex decision logic and provide clinically actionable information to the treating clinician. As such, CDS allows clinicians to focus on caring for patients rather than interpreting complex WGS information, for which they are not traditionally trained to do. Likewise, CDS that provides clear, clinically actionable recommendations derived from WGS information will allow clinicians, even those with minimal training in genetics, to harness WGS information and improve the care of their patients. Moreover, when widespread use of WGS has outpaced the capacity of available genetics professionals, CDS will be able to meet such demands on a widespread scale [19]. This is not to say that genetic professionals will be replaced by CDS, but rather that CDS can manage the common, routine applications of WGS information while allowing genetics professionals to focus their expertise and effort on novel clinical applications of WGS information.

### 3.2. CDS Best Practices

While CDS offers a potential solution to overcome the clinical barriers to WGS adoptions, it is important to consider that not all CDS interventions are successful. Indeed, a systematic review showed that CDS interventions only improved clinical performance about two-thirds of the time [65]. Through practical experience and systematic reviews, researchers have identified important features that contribute to a CDS system being effective. Bates *et al.* summarized their experiences implementing CDS with ‘Ten Commandments for Effective Clinical Decision Support,’ summarized in Table 1 [66]. Kawamoto *et al*. found that computer-generated CDS interventions which are provided automatically during clinical workflow, at the time and location of care, and as care recommendations rather than assessments, are successful more than 90% of the time. This same study found that if any of those key factors were missing, the CDS intervention was successful less than 50% of the time [67]. When developing CDS for WGS, it will be necessary to adhere to these best practices so that the developed CDS solutions have the greatest chance of being successful. Indeed, if such features are not incorporated into CDS interventions for WGS, it will run the risk of failing simply because basic best practices for CDS implementations are not adhered to.

### 3.3. CDS for WGS

CDS for WGS will need to integrate into the clinical workflow and seamlessly provide support at the location and time of decision making, in a manner consistent with best practices. Ideally, CDS would be provided automatically at the time of care within the workflow of the clinician’s EHR or other primary health information systems, such as computerized provider order entry (CPOE) systems. Indeed, clinicians should not be required to use a separate CDS application for WGS information nor even have to initiate the CDS within their primary clinical information system [68]. Moreover, the automatic CDS recommendations could be provided in such a way that the clinician end user would not even need to know that WGS information was being used to generate the recommendation. Preferably, CDS capabilities developed for WGS would not be separate from non-WGS CDS. Rather, WGS information should just be another source of information available for CDS.

**Table 1:** Ten Commandments for effective clinical decision support by Bates *et al*. [66].

Speed is Everything Anticipate Needs and Deliver in Real Time Fit into the User’s Workflow Little Things Can Make a Big Difference Recognize that Physicians Will Strongly Resist Stopping Changing Direction is Easier than Stopping Simple Interventions Work Best Ask for Additional Information Only When You Really Need It Monitor Impact, Get Feedback, and Respond Manage and Maintain Your Knowledge-based Systems

## 4. Potential Clinical Applications of CDS for WGS Information

Genetic information can have many applications in health care. Here, to further make the case for CDS for WGS information, we describe several clinical use cases in which genomic information can be used to guide care. In each example, we propose how automatic CDS leveraging WGS information might be integrated within the clinical workflow. While this is not a comprehensive list, it illustrates various examples of genome-enabled CDS applications at the point of care.

### 4.1. Clinical Diagnosis

Genetic testing is traditionally used to confirm or rule out a diagnosis during the differential diagnosis process [69]. Often, however, clinicians may not know a relevant genetic test is available to support their decision making process. Indeed, some patients often wait months to years to receive an accurate diagnosis, even after seeing several specialists [70]. With a patient’s WGS information readily available, an accurate diagnosis can be reached faster [71,72]. However, for this to happen, it is important to make relevant genetic information easily accessible and reviewable to clinicians at the point of care. While working up a diagnosis, a clinician may not know or be aware of all known genes associated with particular symptoms. At the very least, clinicians should have a simple list of genes containing known pathogenic or likely-pathogenic variants, disease names, and associated phenotypes which the clinician can refer to during the differential diagnosis process. Based upon our current understanding of the human genome, the list of clinically relevant variants will be relatively short (hundreds) [73]. Thus, clinicians could review this information and match it to the patient’s phenotypes; the list should also have the option to view genes containing VUS. Furthermore, disease-causing variants could be automatically added to the EHR’s problem list.

Medical geneticists often search a genetics knowledge base like Online Mendelian Inheritance in Man (OMIM) using phenotypes to identify potentially disease-causing genes for which they could order a genetic test to assess the genotype [74]. Ideally, with the patient’s genome readily available, CDS capabilities integrated with the EHR could automatically perform an ‘OMIM-like’ search on the patient’s genome for candidate gene variants based on phenotypes documented in the EHR. For example, for a child presenting at a pediatric clinic with deafness and heterochromia, which is recorded in the EHR problem list, a CDS system could automatically search the patient’s genome (assuming it is already available) for potential pathogenic variants in genes associated with the presented phenotypes. In this case, if a pathogenic mutation was identified in the patient’s *PAX3* gene, a recommendation can be provided to add Waardenburg Syndrome, a rare genetic disorder, to the problem list [75]. Furthermore, the CDS system could generate a referral to a clinical expert specializing in hereditary deafness who is also covered by the patient’s insurance. While these capabilities could be available in stand-alone genome management products, to be clinically effective they must be integrated within the EHR and clinical workflow.

### 4.2. Disease Risk Assessment

Diseases can be associated with a number of risk factors. Thus, using genetic testing to estimate disease risk is an important aspect of predictive medicine. A gene variant’s influence on disease can range from a slight increase in disease risk to a certainty of future disease onset. With genetic information available, the risk for certain diseases can be deduced, which can then lead to preventative and risk-reducing actions for the patient. To illustrate, women with mutations in the *BRCA1* or *BRCA2* genes have a 50%–80% chance of developing breast cancer in their lifetimes [76]. Knowing this information beforehand can allow women to take risk-reducing actions such as increased screening and prophylactic mastectomy. Unfortunately, as a result of the shortcomings of single gene tests [13], it is estimated that only 5% of women with *BRCA* mutations have been identified with genetic testing [77].

With WGS information readily available, a genome-enabled CDS system could systematically assess the patient’s WGS and clinical information and provide disease risk estimations and risk-reducing recommendations. Such a capability would alleviate the need for clinicians to estimate disease risk on their own. For example, the presence of a pathogenic variant in the *BRCA1* gene (or other genes associated with breast cancer) in a pre-menopausal woman not desiring to have a prophylactic mastectomy could trigger a CDS system to pre-populate an order for more frequent mammograms and to prescribe a selective estrogen receptor modulator, such as tamoxifen. Also, as described above, elevated risk for a disease could automatically be populated on the EHRs problem list. There are a number of stand-alone CDS solutions that provide risk assessment and recommendations for *BRCA* gene mutations [78,79]. However, an ideal scenario would be for such solutions to automatically leverage WGS information and be tightly integrated with the EHR so recommendations are provided within the clinical workflow.

### 4.3. Reproductive Carrier Screening

Related to one’s own disease risk assessment is reproductive carrier screening. Hundreds of congenital disorders are caused by the inheritance of gene variants by one or both parents. Carrier screening is a genetic test that can identify the presence of a disease-causing genetic variant in one or both parents. With this knowledge, it is possible to estimate the risk of having a child affected with a genetic disease, allowing parents to make more informed reproductive decisions. For example, cystic fibrosis is a recessive newborn genetic disorder affecting one in 3,500 births in the U.S. [80]. If both parents are discovered to be carriers for *CFTR* mutations, they can opt for adoption or preimplantation genetic diagnosis to reduce their chances of having a child affected with cystic fibrosis.

Again, CDS could provide support by automatically assessing the patient’s genome to assess their genetic carrier status, and referring the patient to a reproductive specialist or genetic counselor if he or she is considering reproduction. Of note, with autosomal recessive genetic diseases, one parent’s genome is typically only ‘half the equation’; both parental genomes are required to accurately predict disease risks. One could therefore envision an inheritance risk assessment CDS application within the EHR that is able to access *both* parental genomes and provide recommendations only when a risk of having an affected child is present. Similar features are available from commercial laboratory prenatal genetic testing companies such as Counsyl [81]. However, with WGS information readily available for point-of-care CDS, such capabilities could be managed directly by CDS and results presented within the EHR, without the need for another genetic test.

### 4.4. Pharmacogenomics

After a diagnosis has been made, genomic information can be used to guide appropriate therapy and accurate drug dosing. A commonly used example for pharmacogenomics is the use of the anticoagulant warfarin (Coumadin) to prevent thrombosis. Warfarin is metabolized by enzymes derived from the *VKORC1* and *CYP2C9* genes [82]. Variants in these genes can cause patients to be rapid metabolizers of the drug, thereby causing standard dosing regimens to be ineffective. Alternatively, variants can cause patients to be slow metabolizers of the drug, resulting in the drug persisting in the blood longer than expected and accumulating to toxic levels when standard therapeutic doses are administered. By assessing for such gene variants prior to drug therapy, the clinician can reach an optimal therapeutic dose faster while also avoiding adverse events or ineffective treatment regimens, saving lives and unnecessary costs.

Already widely deployed within clinical workflows are drug-drug, drug-allergy, and drug-condition interaction checking within CPOE systems to prevent adverse drug events. These existing CDS capabilities could potentially be extended with WGS information to support drug-gene interaction checking. When a drug like warfarin is prescribed, in addition to checking patient-specific information such as age, weight, and other medications, the CDS rule could also automatically assess the patient’s genome for variants in related genes, such as *VKORC1* and *CYP2C9*, and alert the ordering clinician to any potential complications. Ideally, such a CDS system will not just check for adverse interactions but also provide an optimized drug dose recommendation to the prescribing clinician based on available genetic information and pertinent clinical information [83]. For CDS to be most effective in guiding pharmacogenomics, it needs to be provided within the workflow of the clinician, ideally at the point of order entry. It cannot be expected that a clinician will manually review a patient’s genome for relevant genes variants and then use a stand-alone CDS application, like www.WarfarinDosing.org, every time a drug is prescribed [84]. Some EHR-integrated pharmacogenomics CDS capabilities have been implemented and investigated by researchers at Vanderbilt and St. Jude Children’s Hospital [17,85]. A future step in this research would be to enable the CDS to leverage WGS information and to use CDS capabilities that can be scaled to other institutions.

### 4.5. Nutritional Genomics

The same enzymes that are involved with drug metabolism are also involved in nutrient metabolism. In fact, these enzymes originally evolved for diet; it is only recently in our evolutionary history that clinical medicine has leveraged these enzymes for therapeutics. As such, like pharmacogenomics, nutritional considerations can be personalized based on genetics information. Given that nutrition impacts health, it is essential that clinicians manage nutritional variability caused by genetics. For example, choline, an essential nutrient, is known to be affected by a common variant in the *MTHFD1* gene [86]. People with this variant, particularly pregnant women, need to eat foods rich in choline to avoid adverse consequences of choline deficiency such as neural tube defects in unborn fetuses [87]. Like previous examples, genome-enabled CDS can assess a patient’s genome for such variants and notify the clinician and/or the patient of this risk and make recommendations on how to maintain a diet high in choline.

## 5. Future Direction

A 2012 systematic review [14], as well as more recent manuscripts published after the search period covered by the review describe CDS systems that leverage genetic information [17,18]. With only a few exceptions, these systems primarily leverage a single or a few genes and are typically not automatically integrated within the clinical workflow of the EHR. Nevertheless, these examples represent necessary and important steps toward an ideal CDS solution for WGS information. However, for CDS capabilities to fully meet the demands of WGS information, a number of challenges must be addressed. As such, these challenges will require new CDS approaches that are able to support the unique demands of WGS information. Indeed, a significant amount of work need to be done before WGS information is efficiently incorporated into busy clinical settings through CDS [88,89].

### 5.1. Challenges to Overcome

Several challenges will need to be addressed for the vision of CDS for WGS information to be realized. For example, our understanding of the human genome and its implication on health is still relatively nascent. Research into the genetic contribution to disease has been limited by our ability to leverage a sufficient amount of genetic information and high-quality phenotypic information [90]. The combination of falling genome sequencing costs and EHRs becoming better at representing structured phenotypic information will improve researchers’ ability to identify disease-causing genetic variants on a large scale [91]. These new discoveries are anticipated to lead to many important, clinically relevant recommendations that can be used to improve clinical care. Furthermore, as the genomics knowledge base continues to grow, recommendations will continue to change and evolve with new knowledge. Thus, it is important that CDS be a conduit through which such discoveries can be efficiently translated into clinical care on a widespread scale. 

In addition to the changing clinical knowledge base, an important challenge to WGS-based CDS is the need to maintain a constantly growing and evolving genome variant knowledge base [23]. As described earlier, every patient has many variants in genes associated with diseases. Currently, it is the task of clinical genetic testing laboratories to assign a clinical interpretation (e.g., pathogenic, benign) to the variants detected. Often, however, many interpretations are variants of unknown significance (VUS) because not enough information is known about the variant identified. Although it is the responsibility of the laboratory to notify clinicians to changes in variant interpretation, this can become a daunting and uncompensated task for laboratories to manage because a significant portion of variant interpretations will need to be changed over time. Furthermore, testing laboratories often use their own proprietary repository of variant interpretations and/or many independently-managed gene or disease-specific variant knowledge bases to help make variant interpretations. These variant knowledge bases may only represent a subset of all known variants. Indeed, a centrally-managed gene variant knowledge base would provide great value to improving genome variant interpretation. It is important to note that two large federally-funded efforts, ClinVar and ClinGen, seek to create large publically-available gene and variant knowledge bases to help facilitate gene variant interpretation in the future [92]. However, as these initiatives have just started, it may still be a few years before the full potential of these resources are realized for CDS [93].

Beyond the challenges related to the genome are challenges related to health IT infrastructures that CDS capabilities will be dependent upon. Traditionally, it has been difficult to integrate EHR systems with most third-party CDS applications [94]. While Meaningful Use requirements are making it possible to do more with EHRs, these systems are still challenging to change or to integrate external applications. Therefore, it will be important for CDS capabilities for WGS information to leverage the available features of EHRs, many of which are being made available as a result of Meaningful Use. Furthermore, many CDS capabilities currently available within EHRs are not scalable beyond the institutions at which they are created [95]. Given the time and effort needed to create CDS rules and the potentially extensive list of CDS interventions that could leverage WGS information, the limited scalability of CDS solutions currently available in EHRs will not be sufficient for meeting the full potential of WGS-based CDS. Indeed, current efforts are underway to develop Meaningful Use requirements for EHRs to support service-based CDS capabilities [96]. Without these new CDS service capabilities enabled for EHRs, it will be challenging to support CDS for the WGS on a widespread scale with current CDS capabilities [62,97].

### 5.2. Proposed Solution

**Figure 1:**
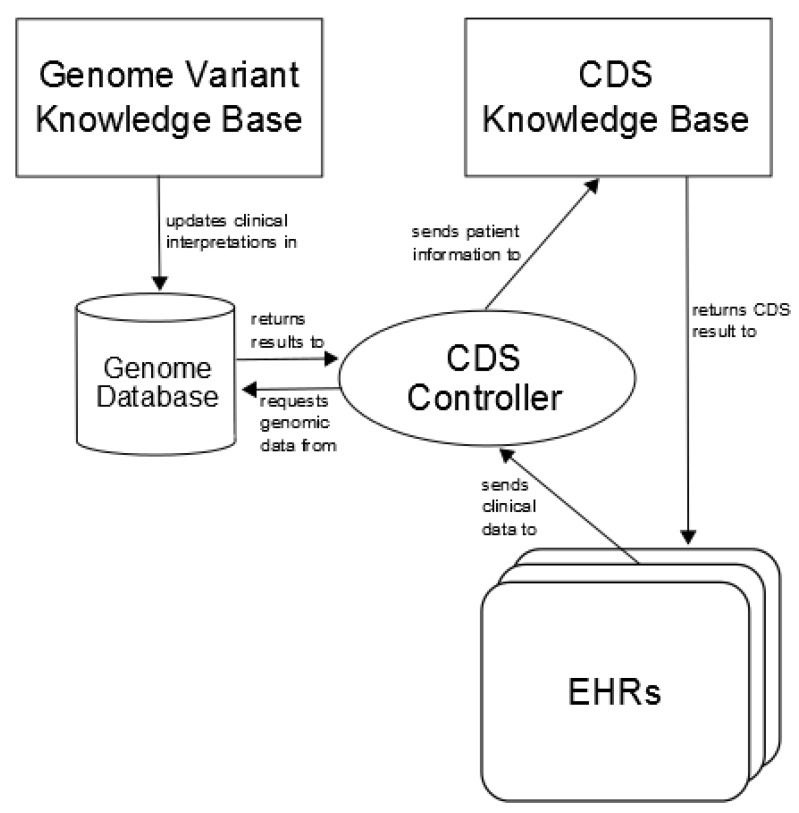
A graphical representation of a proposed scalable clinical decision support (CDS) architecture that can leverage whole genome sequencing (WGS) information.

Given the possibility of leveraging service-based CDS capabilities enabled by Meaningful Use, a standards-based and scalable CDS solution could be developed for WGS information [19]. Given the breadth and complexity of the human genome and the rapidly growing knowledge base, it is unlikely that any single EHR vendor or health care organization will be able to fully manage genomic capabilities on its own. Thus, achieving effective CDS for WGS information will likely require the coordination of several independent services or entities managing specific tasks. See Figure 1. Independent services components that would need to be coordinated could include the EHR, a genome database, variant knowledge base, a CDS knowledge base, and a CDS controller.

In such a scenario, when a CDS service request for WGS information is initiated by an EHR, the request with standardized, structured clinical information document, such as the Health Level 7 Virtual Medical Record (vMR) or Consolidated Clinical Document Architecture (C-CDA), could be sent to the CDS controller which parses the received clinical document into the data required for the rule evaluation that is being requested. The CDS controller would also identify which genetic information is required and submit a query to a genome database for the patient’s genetic information and variant interpretation at a particular loci. The genome database, which stores patients’ genetic information and interpretations, could have its variant interpretations updated and maintained by a separate genome variant knowledge base such as ClinVar. With the most up-to-date clinical interpretation and genetic information returned to the CDS controller, the full set of patient information needed for the CDS evaluation can be sent to and processed by the CDS knowledge base. The WGS-enabled CDS result is then returned to the EHR for presentation within the clinical workflow at the point and time of care. 

Such a solution could allow CDS for WGS to be implemented on a widespread scale with the most accurate and up-to-date information available at any given time. Moreover, as rules could be developed and used by many organizations, the economies of scale to implement new CDS recommendations for WGS information is much lower than if each organization attempted to develop and deploy the same capabilities on their own. Importantly, this approach is aligned with current and future EHR capabilities to support service-based CDS, as anticipated by Meaningful Use Stage 3 requirements. While this infrastructure is largely theoretical construct at this point, efforts are currently underway to develop and validate this proposed CDS architecture approach for WGS information. Nevertheless, before such as architecture can be implemented in a clinical setting on a widespread scale, several considerations need to be resolved. For example: What genetic information is sufficient and necessary for CDS? Who manages and controls each component of the proposed architecture? How can several independent components be coordinated to promote efficiency? Furthermore, it should also be noted that it is yet to be determined if this proposed architecture can meet all needs of WGS information. Indeed, there may be scenarios where a different architecture would be better for certain use cases. This will be determined through continued research and development on CDS capabilities for WGS information.

Finally, clinicians and health IT vendors need to be aware of the coming deluge of genomic information to the clinic so they can be prepared to respond accordingly. Researchers already grapple with overwhelming amounts of genetic information. It is well known by genetics experts that the WGS technologies currently being used in research settings will soon be available to everyday clinicians. Health care organizations and health IT vendors need to be proactive in developing clinical information systems that have the capacity to leverage the WGS in an effective manner. It will likely be impossible for any single health care organization or health IT vendor to manage and support all aspects of genome interpretation and CDS capabilities. As such, health care organizations may need to leverage third party CDS providers, and EHRs need the capacity to support distributed computing architectures, like the one just described, that are capable of integrating external CDS capabilities. 

## 6. Conclusions

We anticipate that WGS capabilities will eventually be routinely available for clinical care. However, without appropriate support, WGS information will likely overwhelm clinicians because of current laboratory reporting methods, the complexity of genetic information, the limited proficiency in genetics by most physicians, and the lack of genetics professionals. However, CDS capabilities can overcome these barriers and increase the likelihood that WGS information can be used effectively for clinical care. Nevertheless, it will be essential that CDS be provided within the clinical workflow and at the point of care in the EHR according to established CDS best practices. We described several clinical use cases using WGS information and described how CDS could be provided within the EHR to support these clinical use cases. Key next steps will be to design and develop a scalable CDS framework capable of leveraging complex WGS information on a widespread scale.
